# Incidence of Acute Kidney Injury in Patients with Chronic Renal Insufficiency: Transcatheter versus Surgical Aortic Valve Replacement

**DOI:** 10.1155/2019/9780415

**Published:** 2019-04-23

**Authors:** Michael Catalano, Dishen Lin, Hugh Cassiere, Nina Kohn, Bruce Rutkin, Greg Maurer, Jacinda A. Berg, Jaclyn Jahn, Rick Esposito, Alan Hartman, Pey-Jen Yu

**Affiliations:** ^1^Division of Cardiovascular and Thoracic Surgery, Zucker School of Medicine at Hofstra/Northwell, 300 Community Drive, 1DSU, Manhasset, NY 11030, USA; ^2^The Feinstein Institute for Medical Research, 350 Community Drive, Manhasset, NY 11030, USA

## Abstract

**Objectives:**

The objective of this study is to determine incidence of acute kidney injury (AKI) associated with transcatheter aortic valve replacement (TAVR) versus surgical aortic valve replacement (SAVR) in patients with preexisting chronic kidney disease.

**Background:**

The incidence of AKI in patients with preexisting renal insufficiency undergoing TAVR versus SAVR is not well described.

**Methods:**

All patients with preexisting chronic kidney disease who underwent SAVR for aortic stenosis with or without concomitant coronary artery bypass grafting or TAVR from 5/2008 to 6/2017. Patients requiring preoperative hemodialysis were excluded. Chronic kidney disease was defined as an estimated glomerular filtrate rate (eGFR) of < 60 mL/min/1.73 m^2^. The incidence of postoperative AKI was compared using the RIFLE classification system for acute kidney injury.

**Results:**

A total of 406 SAVR patients and 407 TAVR patients were included in this study. TAVR patients were older and had lower preoperative eGFR as compared to SAVR patients. Covariate adjustment using propensity score between the two groups showed that SAVR patients were more likely to have a more severe degree of postoperative AKI as compared to TAVR patients (OR = 4.75; 95% CI: 3.15, 7.17; p <.001). SAVR patients were more likely to require dialysis postoperatively as compared to TAVR patients (OR = 4.55; 95% CI: 1.29, 15.99; p <.018).

**Conclusion:**

In patients with preexisting chronic kidney disease, TAVR was associated with significantly less AKI as compared to SAVR.

## 1. Introduction

Aortic stenosis (AS) is the most common valvular heart disease in the United States, with an estimated prevalence of 1.3% among those aged 60-69, 3.9% among those aged 70-79, and 9.8% among those aged 80-89 [1]. If left untreated, symptomatic AS is associated with significant mortality, with an estimated survival of 2 to 3 years [2]. After decades of surgical aortic valve replacement (SAVR) serving as the gold standard for treatment of severe AS, transcatheter aortic valve replacement (TAVR) has emerged as an alternative treatment option for patients at intermediate or higher risk for SAVR.

There is a strong association between chronic kidney disease (CKD) and AS, with up to 75% of patients presenting for aortic valve replacement having some degree of preoperative CKD [3]. Preoperative CKD is an independent predictor of postoperative acute kidney injury (AKI) [4–9]. Patients who develop AKI following SAVR and TAVR have a significantly longer hospital length of stay and higher 30-day and 1-year mortality rate as compared to patients who do not develop AKI [4, 7, 9–11]. Previous studies assessing the risk of postoperative AKI in patients who underwent TAVR as compared to SAVR have provided disparate results [8, 12–15]. The majority of such studies have been limited by inconsistencies in methodology and the lack of standardization of the definition of postoperative AKI.

The purpose of this study is to determine the incidence of AKI, as defined by the risk/injury/failure/loss/end-stage (RIFLE) staging system, in patients with preexisting stage III or higher CKD undergoing TAVR versus SAVR.

## 2. Methods

This study was conducted with the approval of the Northwell Health System Institutional Review Board. As this is a retrospective study utilizing deidentified data that was collected for the New York State and Society of Thoracic Surgeons (STS) databases, specific waiver of the need for individual patient consent was granted by the Institutional Review Board.

All patients with preexisting stage III or higher chronic kidney disease who underwent SAVR for aortic stenosis with or without concomitant coronary artery bypass grafting 2008–2012 or TAVR from 2012 to 2017 were included in the study. Patients who underwent SAVR after the full implantation of the TAVR program in 2012 were excluded to minimize any potential selection bias between the TAVR and SAVR cohorts. Stage III or higher chronic kidney disease was defined as a preoperative estimated glomerular filtrate rate (eGFR) of <60 mL/min/1.73 m^2^. eGFR was calculated using the Chronic Kidney Disease Epidemiology Collaboration (CKD-EPI) equation based on patient age, gender, race, and creatinine [16]. Baseline eGFR was defined by the preoperative creatinine that was the closest to the time of surgery. Patients requiring preoperative dialysis were excluded from the study. The following preoperative data were collected for each patient: age, gender, race, comorbidities (cerebrovascular disease, diabetes, hypertension, dyslipidemia, peripheral vascular disease, congestive heart failure, and moderate or severe chronic obstructive pulmonary disease), body mass index, ejection fraction, hematocrit, preoperative aspirin use, preoperative intra-aortic balloon pump, reoperation, and eGFR. Postoperative variables collected included eGFR, length of stay, discharge location, and dialysis status.

The clinical endpoints were AKI, evaluated using the RIFLE staging system, and postoperative dialysis status. The eGFR at peak postoperative creatinine was used to determine degree of AKI as defined by the RIFLE classification [17]. As suggested by Englberger et al., patients requiring postoperative acute renal replacement therapy were included in the failure class to improve the predictive value of the classification system [18]. As patients had variable volume status and exposure to diuretics perioperatively, urine output was not used in the determination of AKI.

All statistical analyses were performed using SAS version 9.4 (SAS Institute Inc., Cary, NC). Data analysis was performed retrospectively. Propensity scores were calculated using logistic regression, where TAVR (versus SAVR) was the outcome of interest. Factors included in the model were age, gender, race, cerebrovascular disease, diabetes, hypertension, dyslipidemia, peripheral vascular disease, preoperative congestive heart failure, presence of chronic obstructive pulmonary disease, body mass index, ejection fraction, hematocrit, preoperative aspirin use, preoperative intra-aortic balloon pump, reoperation, and preoperative eGFR. The propensity score was included as a covariate in the analyses for postoperative renal failure, need for hemodialysis, and 30-day morality. For renal failure (as measured by RIFLE stage), ordinal logistic regression was used to examine the association between renal failure and procedure. For need of hemodialysis and 30-day mortality, logistic regression was used to examine the association between that outcome and the procedure. Patients who were discharged with a hospital length of stay < 2 days were excluded from the analyses for renal failure and need for dialysis since it was not possible to observe renal failure in that time frame. However, these patients were included in the analysis of 30-day mortality. For each continuous factor included in the calculation of the propensity score, the Mann-Whitney test was used to examine the association between that factor and the procedure. For each categorical factor included in the calculation of the propensity score, the chi-square test was used to examine the association between the factor and procedure.

## 3. Results

Preoperative characteristics for 407 patients with CKD undergoing TAVR and 406 CKD patients undergoing SAVR are listed in Table 1. TAVR patients were older, had a lower body mass index, and had a higher preoperative ejection fraction than SAVR patients. They were also more likely to have preexisting dyslipidemia and preoperative aspirin use, and they were more likely to be undergoing reoperation and less likely to have COPD. TAVR patients were also more likely to be African-American and have a lower preoperative GFR than SAVR patients (45.8 mL/min/1.73 m2 vs. 47.4 mL/min/1.73 m2, p < 0.012). There was no significant difference between patient groups in gender and other comorbidities (including diabetes, hypertension, CHF), hematocrit, and use of intra-aortic balloon pump. The SAVR patients had lower Society of Thoracic Surgeons Predicted Risk of Mortality (STS-PROM) as compared to TAVR patients with a median PROM of 5.0% (interquartile range 3.0-9.0) for SAVR versus 7.7% (interquartile range 5.1-10.5) for TAVR patients (p<0.001).

**Figure 1:**
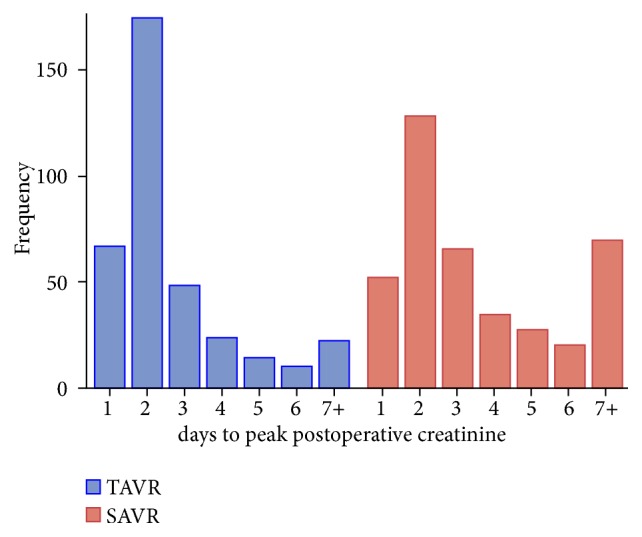
Time to the lowest postoperative eGFR, TAVR vs. SAVR.

Eight (1.97%) of the patients in the TAVR cohort and 12 (2.96%) of the patients in the SAVR cohort died within 30 days of operation. This difference was not significant (OR: 2.38, 95% Confidence Interval (CI): 0.85, 6.64; p<0.10). As the majority of both TAVR and SAVR patients reach their lowest eGFR at postoperative day 2 or greater (Figure 1), patients who had length of stay < 2 days were excluded from postoperative AKI analysis. Eight TAVR patients and 4 SAVR patients were discharged with a length of stay < 2 days and, so, were excluded from these analyses. The postoperative outcomes of the remaining 399 TAVR patients and 402 SAVR patients are shown in Table 2. Patients undergoing TAVR were significantly less likely to experience postoperative AKI, with 11.78% of patients experiencing any RIFLE classification of AKI as compared to 38.30% of patients undergoing SAVR. The percent of TAVR patients with Risk, Injury, and Failure classification of postoperative AKI was 9.52%, 1.00%, and 1.25%, respectively. In comparison, the percent of SAVR patients with Risk, Injury, and Failure classification of postoperative AKI was 27.36%, 7.71%, and 3.23%, respectively. Covariate adjustment using propensity score between the two groups showed that SAVR patients were more likely to have a more severe degree of postoperative AKI as compared to TAVR patients (OR: 4.75; 95% CI: 3.15, 7.17; p <.001). SAVR patients were more likely to require dialysis postoperatively as compared to TAVR patients (OR: 4.55; 95% CI: 1.29, 15.99; p <.018). Inclusion of STS-PROM into the calculation of the propensity score used for covariate adjustment did not qualitatively change the above results with SAVR patients remaining to be at an increased risk for postoperative AKI (OR: 4.75; 95% CI: 3.04, 7.41; p <.001) and dialysis (OR: 5.31; 95% CI: 1.31 21.54; p <.02).

The median cardiopulmonary bypass time for the SAVR patients was 144 minutes (interquartile range 114–182). There was no association between bypass time and AKI (OR: 1.00; 95% CI: 1.00, 1.01; p=0.20). The median contrast volume for the TAVR patients was 69cc (interquartile range 50 – 100). There was no association between contrast volume and AKI (OR: 1.00; 95% CI: 0.99, 1.01; p=0.68). There was no association between preoperative eGFR and postoperative AKI in SAVR patients (OR for a 10-point decrease in preoperative eGFR: 1.21, 95% CI: 0.99, 1.47; p<0.059). Similarly, there was no association between preoperative eGFR and postoperative AKI in TAVR patients (OR for a 10-point decrease in preoperative eGFR: 0.851, 95% CI: 0.64, 1.14; p<0.275).

The timing to maximal postoperative AKI as manifested by the lowest postoperative eGFR was reached earlier for TAVR patients as compared to SAVR patients (Figure 1). TAVR patients reached the lowest postoperative renal function at a median of 2 days (interquartile range 1.0–3.0 days) as compared to 3 days (interquartile range 2.0–5.0 days) for SAVR patients (p <.001). Interestingly, although 38.30% of patients undergoing SAVR developed AKI at some point during their postoperative course, the majority of these patients improved their renal function during their index admission with only 8.46% of patients having a decrease in eGFR of greater than 25% from baseline at discharge.

Additionally, TAVR patients were significantly more likely to experience shorter hospital stays (median stay 4.0 days (95% CI: 3.0, 4.0) vs. 8 days (95% CI: 7.0, 8.0), p <.001) and be discharged to home (81.05% vs. 46.17%, p <.001).

## 4. Discussion

This single-center retrospective study of 813 TAVR and SAVR patients with stage III CKD demonstrated that SAVR patients are significantly more likely to develop postoperative AKI as defined by the RIFLE criteria and require postoperative hemodialysis as compared to TAVR patients.

AKI after aortic valve replacement is associated with significantly worse patient outcomes in terms of mortality, length of stay, and hospital costs [4, 7, 9, 11]. The reported incidence of AKI after aortic valve replacement has been widely varied, ranging from 3.4–43% for patients undergoing SAVR and 3.4–57% for patients undergoing TAVR [10]. This variability is largely secondary to the heterogeneity in the patient population and inconsistencies in the definition of AKI between different studies. A number of previous studies have compared the risk of AKI in patients undergoing TAVR versus SAVR with disparate results. A propensity-matched study by Results of the Placement of Aortic Transcatheter Valves (PARTNER) trial showed no significant difference in postoperative AKI in high-risk patients randomized to TAVR or SAVR [19]. Similarly, Thongprayoon et al. found no significant difference in postoperative AKI, major adverse kidney events, in-hospital mortality, or 6-month mortality between TAVR and SAVR cohorts [8, 20]. In contrast, a continuous propensity score analysis of patients with preoperative CKD undergoing aortic valve surgery by Bagur et al. found that the incidence of postoperative AKI was lower in the TAVR cohort compared to the SAVR cohort (9.2% versus 25.9%, p = 0.001) [21]. However, the results of that study were based on a single determination of eGFR 48 hours postoperatively, which may not capture the true incidence or degree of postoperative AKI. A similar study by D'Errigo et al. compared the incidence of AKI using the Acute Kidney Injury Network (AKIN) definition in propensity matched patients with CKD undergoing TAVR and SAVR [12]. They reported higher incidence of postoperative AKI in patients undergoing SAVR as compared with patients undergoing TAVI (48.9% versus 35.8%, p=0.04) but were unable to demonstrate differences in rates of de novo dialysis. The use of AKIN criteria, however, has been shown to overdiagnose AKI in patients after cardiopulmonary bypass [18]. Doshi et al. conducted a retrospective database analysis using the National Inpatient Sample (NIS) to assess the impact of TAVR and SAVR on outcomes in patients with advanced renal disease, and they found a significantly reduced rate of AKI in TAVR (33%) compared to SAVR (50%). This analysis is limited, however, by the use of nonstandardized diagnostic codes to identify preoperative renal function and detect postoperative AKI [13].

Our study is one of the largest studies to use standardized criteria for AKI to compare the incidences of AKI in patients undergoing TAVR versus SAVR. As preoperative renal function is a strong predictor of postoperative AKI [22–24], we included only patients with preoperative CKD stage III or higher, representing the highest-risk patients for the development of postoperative AKI. The divergent results of prior studies looking at the incidence of AKI in patients undergoing TAVR and SAVR can be in part attributable to the heterogeneity of the definitions of AKI used in the studies, the most common used being the RIFLE, AKIN, and Kidney Disease Improving Global Outcomes (KDIGO) classification. The Society of Thoracic Surgery utilizes the RIFLE classification in its definition of postoperative AKI, while the Valve Academic Research Consortium (VARC) recommends the use of AKIN definition for patients undergoing TAVR [25]. While RIFLE uses preoperative baseline renal function to define postoperative AKI, both AKIN and KDIGO use a moving window of baselines that requires only changes in renal function over any 48-hour period to define AKI. As there is a known physiological decrease in serum creatinine secondary to hemodilution following cardiopulmonary bypass [26], the use of the AKIN and/or KDIGO criteria with its 48-hour moving diagnostic interval can lead to a fourfold higher diagnosis of postoperative AKI in patients undergoing cardiac surgery as compared to the use of the RIFLE criteria [18]. Given the potential for overdiagnosis of AKI in post-cardiopulmonary bypass patients with the use of the AKIN criteria, we elected to use the RIFLE definition for the purpose of this study for a more accurate comparison between TAVR and SAVR patients. Furthermore, as patients requiring aortic valve replacement are generally elderly, eGFR was used in this study instead of creatinine as the use of serum creatinine has been shown to be not sensitive when evaluating renal dysfunction in patients with a relatively lower muscle mass such as in women and/or elderly patients [27]. The use of eGFR was also shown to be more sensitive than serum creatinine for evaluating for the diagnosis of AKI [28].

Our study did not show an association between contrast volume and postoperative AKI in TAVR patients. Although this may be contrary to the known nephrotoxic effect of contrast media on renal function, our result is congruent with other studies that also failed to show a significant association between volume of contrast media and post-TAVR AKI [21, 29–31]. The minimization of contrast volume and the use of renal protective strategies under the guidance of a nephrologist may have diminished the nephrotoxic effect of contrast media on our patients.

There are several limitations to this study that should be acknowledged. First, as with all nonrandomized studies utilizing propensity scores, this study is limited by the assumption that all covariates are accounted for in the propensity score. Secondly, as postoperative AKI was identified by postoperative eGFR during the index admission, decline in eGFR postdischarge would not be accounted for in the classification of AKI. As TAVR patients have a shorter length of hospital stay as compared to SAVR patients, TAVR patients with delayed AKI are less likely to be captured in this study as compared to SAVR patients. However, the observation that TAVR patients reach their nadir eGFR earlier than SAVR patients may help mitigate some of this limitation of this study. Thirdly, the SAVR and TAVR cohorts were not contemporaneous as patients who underwent SAVR after the full implantation of the TAVR program were excluded in this study to minimize any potential selection bias. Although this may introduce confounders secondary to differences in practice over time, this is minimized by the fact that there were no significant changes in the surgeon and intensivist staff who were directing patient care during the study period. Finally, as with all single-center studies, the results of this study may not be generalizable to other institutions.

## 5. Conclusions

Acute kidney injury represents a significant risk for patients with preexisting CKD undergoing aortic valve replacement surgery. TAVR is associated with significantly less AKI and fewer postoperative complications than SAVR in these patients.

## Figures and Tables

**Table 1:** Pre-Operative Characteristics of the Patient Population.

Pre-Operative Characteristics	TAVR (%)	SAVR (%)	P-value
	n = 407	n = 406	
Age	85.0 (80.0-89.0)	80.0 (76.0-85.0)	<.001
Male	194 (47.67)	218 (53.69)	0.09
African-American	18 (4.42)	7 (1.72)	0.03
Cerebrovascular Disease	114 (28.01)	81 (19.95)	0.01
Diabetes	144 (35.38)	156 (38.42)	0.37
Hypertension	366 (89.93)	350 (86.21)	0.10
Dyslipidemia	366 (89.93)	266 (65.52)	<.001
Peripheral Vascular Disease	82 (20.15)	59 (14.53)	0.04
Congestive Heart Failure	172 (42.46)	181 (44.58)	0.50
Chronic Obstructive Lung Disease	63 (15.48)	88 (21.67)	0.02
Body Mass Index	26.8 (24.1-30.8)	27.9 (24.9-31.8)	0.01
Ejection Fraction	60.0 (51.0-69.0)	55.0 (45.0-60.0)	<.001
Hematocrit	35.6 (32.4-39.0)	36.0 (33.0-39.5)	0.34
Pre-Operative Aspirin Use	275 (67.57)	192 (47.29)	<.001
Intraaortic Balloon Pump	2 (0.49)	7 (1.72)	0.11
Reoperation	81 (19.90)	59 (14.53)	0.04
eGFR	45.8 (35.7-53.0)	47.4 (38.8-53.8)	0.01

Median and interquartile range (Q1 - Q3) are given for continuous factors

eGRF = estimated glomerular filtrate rate.

**Table 2:** Post-Operative Outcomes of the Patient Population.

Pre-Operative Characteristics	TAVR (%)	SAVR (%)	Odds Ratio	p-value
	n = 399	n = 402	(95% CI)	
RIFLE Stage			4.75 (3.15-7.17)	<0.001
eGFR <25% decrease	352 (88.22)	248 (61.69)		
R: eGFR decrease >25%-50%	38 (9.52)	110 (27.36)		
I: eGFR decrease >50-75%	4 (1.00)	31 (7.71)		
F: eGFR decrease >75% OR RRT	5 (1.25)	13 (3.23)		
Time to Lowest eGFR (median, IQR)	2.0 (1.0 - 3.0)	3.0 (2.0 - 5.0)		<0.001
Discharged Home	325 (81.05)	181 (46.17)	7.71 (5.17-11.48)	<0.001
Dialysis	4 (1.00)	12 (2.99)	4.55 (1.29-15.99)	0.02

eGRF = estimated glomerular filtrate rate; F = failure; I = injury; IQR = interquartile range; R = risk; RRT = renal replacement therapy.

## Data Availability

The data used to support the findings of this study are available from the corresponding author upon request.
